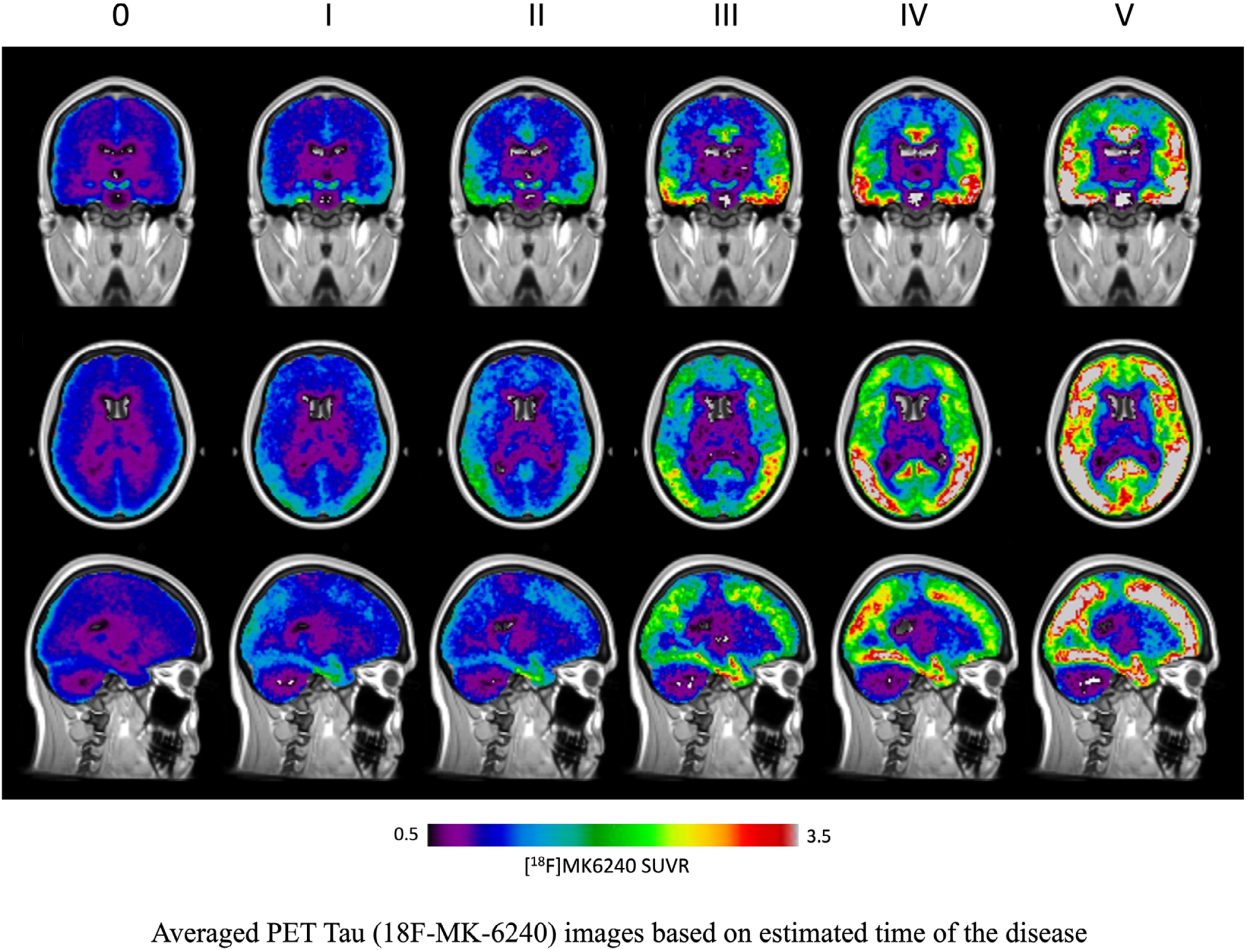# Staging of tau accumulation associated with cognitive decline in AD progression using 18F‐MK‐6240 PET data

**DOI:** 10.1002/alz.092014

**Published:** 2025-01-09

**Authors:** Neda Shafiee, Vladimir S Fonov, Reza Rajabli, Joseph Therriault, Nesrine Rahmouni, Stijn Servaes, Serge Gauthier, Jenna Stevenson, Nina Margherita Poltronetti, Pedro Rosa‐Neto, D Louis Collins

**Affiliations:** ^1^ McConnell Brain Imaging Centre, Montreal Neurological Institute, McGill University, Montreal, QC Canada; ^2^ Douglas Mental Health University Institute, Montreal, QC Canada; ^3^ Translational Neuroimaging Laboratory, The McGill University Research Centre for Studies in Aging, Montréal, QC Canada; ^4^ Translational Neuroimaging Laboratory, The McGill University Research Centre for Studies in Aging, Montreal, QC Canada; ^5^ Centre for Studies on Prevention of Alzheimer's disease (StoP‐AD Centre), Montreal, QC Canada

## Abstract

**Background:**

Alzheimer's disease is characterized by the accumulation of amyloid beta and the formation of tau neurofibrillary tangles (NFTs), leading to irreversible neurodegeneration. The formation of NFTs is believed to follow a stereotypical pattern known as Braak stages. Here, using tau‐PET tracer 18F‐MK‐6240 we aim to analyze patterns of Tau accumulation associated with AD‐related cognitive decline and build an in‐vivo, data‐driven staging system based on longitudinal data, using an estimated latent time of disease onset based on cognitive scores to place all subjects on a common timeline.

**Method:**

We used 18F‐MK‐6240 scans from the TRIAD dataset, including 194 cognitively normal, 99 mild cognitive impairment and 77 with Alzheimer’s disease dementia. We used a trajectory model (Kühnel et al. 2021) to align patients based on their longitudinal cognitive scores along a continuous latent disease timeline. ADAS‐cog‐13, CDR‐SB and MMSE were used simultaneously to estimate time‐shifts for each subject. As there were not enough longitudinal time‐points in the TRIAD dataset to directly apply this method, we first applied the method to the full ADNI dataset and then used a nearest‐neighbour technique to impute the disease offset for TRIAD subjects from the closest 38 subjects in the ADNI cohort. (n=38 was found to be optimal through cross‐validation within ADNI.) This supervised imputation model used baseline cognitive test scores (MMSE and CDR‐SB) along with the age of participants to impute their latent disease onset.

**Result:**

We defined 5 2‐year windows on the 10‐year span of the estimated latent disease offset timeline from 4y before onset and up to 6y afterwards. Tau PET scans for subjects within each window were averaged, resulting in 5 average Tau templates, in addition to an initial template generated using amyloid negative normal participants. This staging system depicts the incremental tau accumulation along with the decline in cognition. Medial temporal regions show initial accumulation, starting in transentorhinal and entorhinal cortices, and later stages show full brain involvement.

**Conclusion:**

Using purely data‐driven techniques, this method reveals patterns of tau accumulation associated with cognitive decline. These models will help understand the link between Tau and cognitive decline, even for those subjects that don't fit Braak framework.